# The Impact of Aging on Neurological Diseases in the Elderly: Molecular Mechanisms and Therapeutic Perspectives

**DOI:** 10.14336/AD.2024.1085

**Published:** 2025-11-05

**Authors:** Dan Zhou, Yumeng Lin, Zhongyu Han, Zhuyun Zhang, Le Lin, Shichong Lin, Qianke Yang

**Affiliations:** ^1^Department of Physical Medicine and Rehabilitation, The Second Affiliated Hospital and Yuying Children's Hospital of Wenzhou Medical University, Zhejiang, China.; ^2^School of Smart Health Care (School of Health & Medical), Zhejiang Dongfang Polytechnic, Zhejiang, China.; ^3^Health Management Center, Nanjing Tongren Hospital, School of Medicine, Southeast University, Nanjing, China.; ^4^School of Medicine, Southeast University, Nanjing, China.; ^5^Department of Critical Care Medicine, Shenzhen People’s Hospital, First Affiliated Hospital of Southern University of Science and Technology, The Second Affiliated Hospital of Jinan University, Shenzhen, China.

**Keywords:** Aging, Hallmark, Alzheimer's disease (AD), Parkinson's disease (PD), Huntington's disease (HD), Amyotrophic lateral sclerosis (ALS), Multiple sclerosis (MS)

## Abstract

With the progression of global aging, neurological diseases in elderly individuals have aroused widespread interest among researchers. Imbalances in the homeostasis of neuronal microenvironments, including neural progenitor cells and microglia, are the leading cause of worsening neurodegenerative diseases. The aging of various glial cells can further lead to abnormal functions in the central nervous system (CNS). Recent studies have shown that aging plays a vital role in a variety of degenerative diseases, including Huntington's disease (HD). In this manuscript, we describe the molecular mechanisms of aging, the cellular constitution of the neural microenvironment and the progression of aging in various neurodegenerative diseases, providing new targets and perspectives for the clinical treatment of various neurodegenerative diseases.

## 1. Introduction

Aging continuously occurs in the human body beginning at birth and leads to an increasing risk of death and disease with aging. Two categories of factors affect aging: those that are programmed and those that are related to damage. Several factors contribute to a reduction in telomere length, such as decreases in the levels of growth hormones, changes in the levels of reproductive hormones, and diminished immune responses. Damage-related variables include DNA damage that has not been sufficiently or correctly repaired; the metabolised of cellular waste, environmental toxins or free radicals from normal metabolism; and aging markes,which are the subject of ongoing scientific research. Differences in susceptibility to aging have also been noted.

Three possible scenarios for physiological aging versus actual aging have been identified: “accelerated aging” ( the biological age is older than the chronological age), “normal aging” (the biological age is equal to the chronological age), and “super aging” (the chronological age is older than the biological age) [1].

The cardiovascular and nervous systems are more susceptible to aging than the gastrointestinal system is, according to a the study of cerebral ischemia mechanisms [1]. These findings may be related to these structures in the neurovascular unit (NVU). In the nervous system, the hippocampus, substantia nigra dense, and the ventral region are particularly susceptible to age-related changes compared with other regions [2], and a direct relationship has been observed between the pathology of neurodegenerative diseases and how these parts regions. Notably, autoimmune and metabolic dysregulation are also related to aging. Several interventions for neurodegenerative diseases are available and are based on consideration of these mechanisms of aging and neurodegenerative diseases (e.g., AD, ALS, HD, PD, and MS).

This review aims to present a thorough synopsis and analysis of the interactions between the underlying processes of aging and the pathological features of AD, ALS, PD, MS, and HD. It also systematically outlines the pertinent interventions, including pharmacological treatments and rehabilitation strategies, informed by the pathophysiological mechanisms of these diseases. Furthermore, the review provides a forward-looking perspective, exploring potential future directions and innovations in the field.

## 2. Mechanism of aging

In recent years, the academic community has been researching aging hallmarks, which are categorized into three main types: primary hallmarks, antagonistic hallmarks, and integrative hallmarks. Primary hallmarks are considered a significant driving force of aging [3], antagonistic hallmarkers are associated with the compensatory or antagonistic response to aging, and integrative hallmarks are mainly associated with counteracting the cumulative damage caused.

Primary markers of aging include oxidative damage, telomere length shortening, decreased mitochondrial function, and abnormal protein aggregation, all of which are direct causes of aging. Antagonistic hallmarks are factors that can resist senescence and maintain cell and tissue functions, such as autophagy, mitochondrial biosynthesis, and the antioxidant system. These hallmarks can be considered compensatory or antagonistic senescence markers. These processes can be considered compensatory or antagonistic mechanisms of aging. Integrative hallmarks are indicators of cumulative cellular and tissue damage, such as abnormal cell cycle protein expression, increased genomic instability, and a decline in tissue function. These markers are the result of the aging process.

### 2.1 Primary hallmarks

The primary hallmarks are divided into four major categories: genomic instability (the most predominant one), telomere attrition, epigenetic alterations, and a loss of the genome. Epigenetic alterations are related mainly to protein homeostasis.

### 2.1.1 Genomic instability

**Figure 1.**
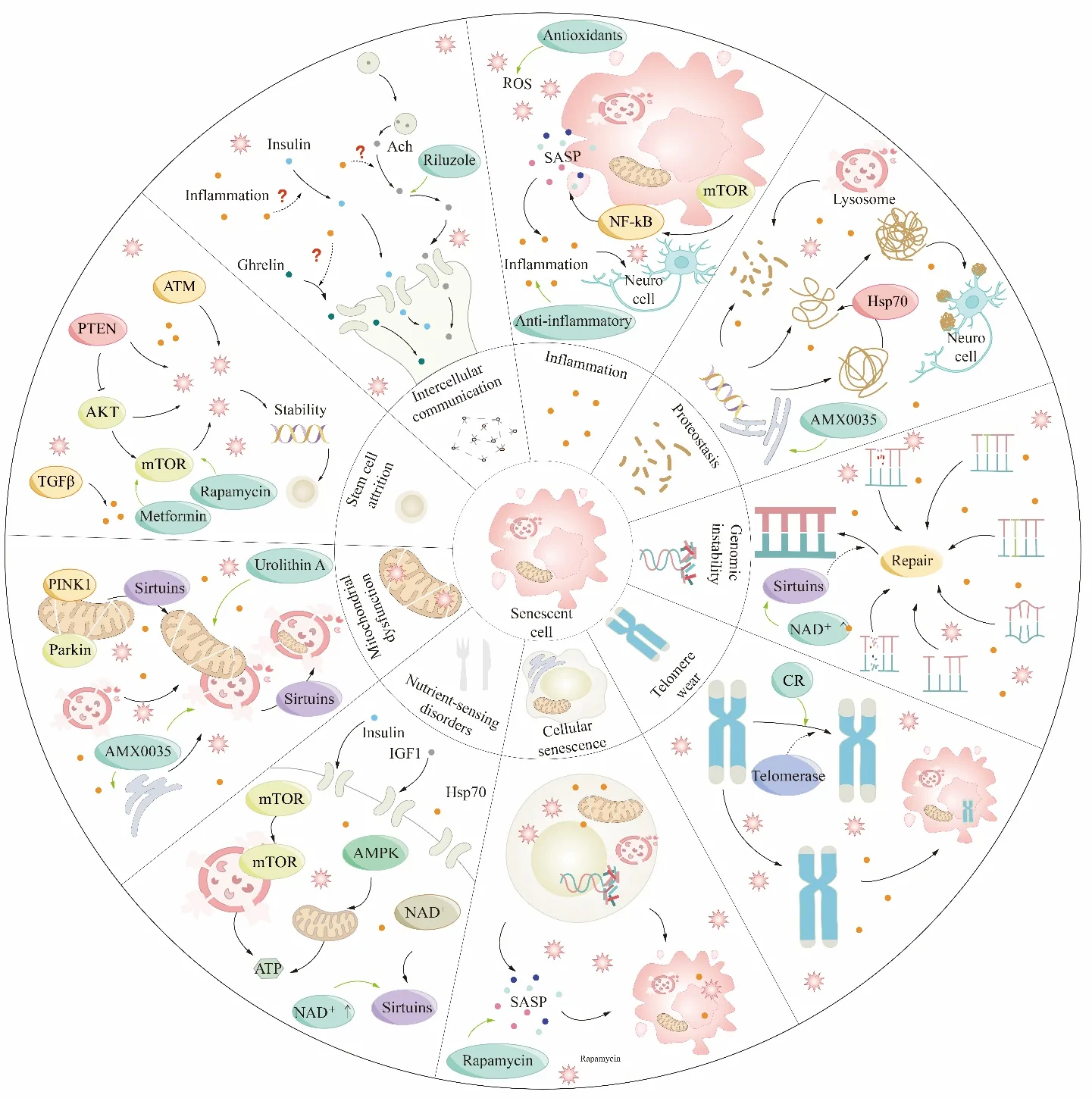
**Related mechanisms of ageing and intervention therapy**. Ageing impacts various cellular functions, including intercellular communication which can lead to a decline in tissue coordination, inflammation that may cause chronic low-grade responses, proteostasis imbalance resulting in protein misfolding and aggregation, genomic instability due to DNA damage and errors in repair mechanisms, telomere wear that shortens DNA ends leading to cellular aging, cellular senescence where cells lose their ability to divide, nutrient-sensing disorders that affect metabolic regulation, mitochondrial dysfunction affecting energy production and cellular respiration, and stem cell attrition which reduces the regenerative capacity of tissues. These interconnected factors contribute to the overall aging phenotype and the increased susceptibility to age-related diseases. Aging implicates a spectrum of cellular degenerations, including declines in tissue coordination, inflammation, oxidative stress, and increased susceptibility to diseases. Proposed interventions target these aspects to delay aging effects: enhancing intercellular communication via NAD^+^ to activate sirtuins; employing anti-inflammatory diets and medications to curtail chronic inflammation; utilizing antioxidants to mitigate oxidative stress and protect cellular integrity; inhibiting the mTOR pathway with Rapamycin and Metformin to slow cellular senescence; optimizing mitochondrial function and energy metabolism through mitophagy and compounds like Urolithin A; mitigating ER stress with agents such as AMX0035 to support protein homeostasis; and implementing CR, particularly intermittent fasting, to extend lifespan by promoting cellular repair and energy conservation. Stem cell activity enhancement improves tissue repair capabilities, countering the regenerative decline of aging. This figure illustrates the integrated approach of these interventions to attenuate the aging phenotype and potentially enhance health span.

Genomic instability mainly refers to DNA damage, the main mechanisms of which include base shedding, double-strand breaks, single-strand breaks, base mismatches, insertions and deletions (Fig. 1) [4]. Oxidative DNA damage caused by endogenous reactive oxygen species (ROS) induces inflammation, accelerates aging, and increases susceptibility to cancer and neurodegenerative diseases [5, 6].

Researchers have shown that neurons in aged mice accumulate large amounts of DNA damage [7] and exhibit elevated expression of proinflammatory molecules [8, 9]. DNA damage results in genomic instability and initiates signalling cascades that spread throughout the cell; additionally, the viability of highly damaged cells is reduced.

Damage to nuclear DNA may affect insulin signaling, DNA and protein methylation and acetylation, DNA repair, and lipid metabolism [10]. Since the electron transport chain, which produces many types of ROS, is close to mitochondrial DNA, damage to its histones accumulates more quickly and efficiently because of the absence of DNA protection in mitochondria. Due to the high replication rate of mitochondrial DNA, damage to mitochondrial DNA can spread more quickly [11].

DNA damages increases the ROS index in the affected brain tissues of patients with neurodegenerative diseases, including AD, PD and ALS [12]. However, the human body has self-repair methods for DNA damage [13], which include direct inversion, mismatch repair, mucleotide excision repair (NER), base excision repair (BER) [14], and DNA double-strand break repair (DSBR).

### 2.1.2 Telomere wear

Telomere wear is closely linked to the aging process [15]. Telomeres are protective caps located at the ends of chromosomes because of the inability of DNA polymerase to fully replicate linear chromosome ends as the cell divides and increases in value, leading to cellular senescence [16], which is related to organismal aging.

Telomere attrition plays a crucial role in the pathogenesis of neurodegenerative diseases by compromising cellular longevity and function. As telomeres shorten with each cell division, they eventually reach a critical length that triggers cellular senescence or apoptosis. In the context of the central nervous system, where many neurons are postmitotic, telomere attrition in glial cells and neural stem cells is particularly significant. Shortened telomeres activate the p53-p21 [17] and p16-pRB pathways [18], leading to cell cycle arrest and the senescence-associated secretory phenotype (SASP) [19]. The SASP contributes to chronic inflammation through the release of proinflammatory cytokines, chemokines, and matrix-degrading proteins. This inflammatory milieu is neurotoxic and can exacerbate neuronal loss. Moreover, telomere attrition in neural stem cells diminishes the regenerative capacity of the brain, impairing neurogenesis in regions such as the hippocampus, which is crucial for memory formation and is severely affected in patients with Alzheimer's disease. Studies have shown that patients with neurodegenerative diseases often exhibit accelerated telomere shortening in various cell types, including leukocytes and neural cells, which is correlated with disease severity and progression.

Telomere wear may cause mental health conditions and cognitive decline, and telomere maintenance is essential for stem cell survival and self-renewal in the brain [20, 21]. For example, the microglia of AD patients have shorter telomeres than those of age-matched controls [22]. Nonneuronal brain cells such as neural stem cells and astrocytes divide more slowly than cells in other tissues do and have slower telomere wear rates than in other cells. Since telomeres are located the ends of chromosomes, their dysfunction may lead to genomic instability [23, 24].

Several observations, such as the association of short telomeres with early death from age-related diseases [25] and the correlation of telomere length with age in various tissues [26-28], support the relationship between telomere wear and senescence. In addition, interventions that can increase the telomere length or delay telomere shortening, such as caloric restriction (CR) [29] or the activation of telomerase [30], have shown the potential to extend lifespan in a variety of organisms and may improve the healthy lifespan.

### 2.2 Antagonistic hallmarks

The antagonistic hallmarks [16] are markers that are activated under certain internal and external conditions and are divided into three main categories: mitochondrial dysfunction, cellular senescence, and nutrient-sensing disorders.

### 2.2.1 Mitochondrial dysfunction

According to the mitochondrial free radical theory of aging, prolonged exposure to ROS can cause oxidative damage and aging of mitochondrial DNA. Nucleic acids, lipids, and proteins can all be damaged by ROS [31]. Since the primary source of these free radicals is the mitochondria, their levels increase with age and are associated with poor mitochondrial integrity.

Maintaining mitochondrial function requires proteasomal degradation, the export of defective proteins from vesicles produced from mitochondria, the fission and fusion of mitochondria, removal by proteases, and the autophagy of mitochondria. The functional properties of mitochondria are associated with their dysfunction. Mitochondrial autophagy is necessary to remove damaged mitochondria, maintain mitochondrial quality control, and lower inflammation. One of the autophagy pathways is the PTEN-induced putative kinase protein 1 (PINK1) -Parkin pathway [32], which is also regulated by FUNDC1, AMBRA1, NIX/BNIP3, MUL1, or BCL-2-L-13 [33]. Mitochondrial dysfunction is a significant potentially contributing contributing to AD [34], PD [35] and ALS [36].

Mitochondrial dysfunction is a central feature in the pathogenesis of many neurodegenerative diseases, given the high energy demands and sensitivity of the brain to oxidative stress. The primary pathways through which mitochondrial dysfunction [31] contributes to neurodegeneration include impaired energy production, increased oxidative stress, and dysregulation of calcium homeostasis. Defects in the electron transport chain, particularly in complexes I and IV, lead to decreased ATP production and increased ROS generation [37]. Excess ROS damage cellular components, including proteins, lipids, and DNA, particularly mitochondrial DNA which lacks protective histones. This process is a vicious cycle in which mitochondrial DNA damage further impairs mitochondrial function. Mitochondrial dysfunction also disrupts calcium homeostasis, leading to excitotoxicity and the activation of apoptotic pathways [38]. In individuals with Parkinson's disease, mitochondrial complex I deficiency is a hallmark feature, that contributes contributing to the selective vulnerability of dopaminergic neurons in the substantia nigra [39].

Researchers have attempted to use medications targeting mitochondrial dysfunction to treat neurodegenerative diseases, such as the use of antioxidant medications, and specific pharmacological interventions for particular diseases are described later.

### 2.2.2 Cellular senescence

An irreversible process known as "cellular senescence" causes cells to cease proliferating and exhibit changes in certain phenotypes, such as chromatin and secretome patterns [27, 40-43]. Senescence includes replicative senescence and stress-induced premature senescence, which represents a state in which cell growth is permanently arrested [44-46], and mainly refers to telomere wear and tear, inflammation, autophagy defects, chronic DNA damage response (DDR) [47], oxidative stress and mitochondrial dysfunction. This process can occur at any life stage from the embryonic stage to adulthood and is characterized by three main features: the loss of the proliferative or regenerative capacity, altered metabolic function and resistance to apoptosis, and the secretion of a range of active pathogenic molecules [48]. The primary function of senescence is to inhibit the growth of damaged cells and activate the immune system to induce cell death. Thus, senescence may be a beneficial compensatory response that helps to remove damaged and potentially carcinogenic cells from tissues. Immune surveillance and phagocytosis eliminate senescent cells, but senescent cells also seem to accumulate with aging [49-51]. This accumulation may be the result of a combination of their increased production and reduced immune clearance. While senescent cells secrete SASP factors [19], aging may be influenced by this proinflammatory secretome (see the "Altered intercellular communication" section) Studies have also revealed found an increase in the number of senescent cells in aged tissues and tissues with chronic age-related diseases [43, 52] as well.

### 2.2.3 Nutrient-sensing disorders

The main idea behind nutrient-sensing dysregulation is that CR inhibits nutrient signaling pathways, which may have neuroprotective effects on human tissues and have been shown in tests to increase the lifespan of a range of animals, including mice. insulin and IGF-1 signalling (IIS) is referred to as the IIS pathway, although important materials and mechanisms involved in food sensing include insulin; insulin-like growth factor 1 (IGF-1), the mechanistic target of IGF-1; mechanistic target of rapamycin (mTOR); and sirtuins [16]; AMP-activated protein kinase (AMPK). AMPK uses high AMP levels to identify low-energy states; sirtuins use high NAD^+^ levels to identify low-energy states; and mTOR is a sensor of increased concentrations of amino acids [53].

Paradoxically, in both mouse models of premature aging and during normal aging, IGF-1 levels decrease [53]. Thus, reduced IIS is a common feature of both physiological and accelerated aging, and a constitutively reduced IIS extends the lifespan. Levels to sense low energy states [1]. Several studies on mTOR, showed that it was effective at extending the lifespan of yeast, worms, and drosophila and mice [54, 55]. AMPK and sirtuins communicate catabolic signals and nutrient deficiency rather than anabolic signals and nutrient enrichment because they operate in an opposite manner to IIS and mTOR. Thus, their upregulation promotes healthy aging. The evidence shows that metformin administration to worms and mice may extend their longevity through AMPK activation [56-58].

Overall, the currently available data strongly support the theory that decreased nutritional signaling prolongs delays longevity, whereas anabolic signaling accelerates aging [59].

### 2.3 Integrative hallmarks

Integrative hallmarks refer to the overarching phenotypes that manifest due to the cumulative and synergistic effects of multiple antagonistic hallmarks of aging. The primary integrative hallmarks are stem cell attrition and altered intercellular communication.

### 2.3.1 Stem cell attrition

One of the most noticeable effects of aging is a reduction in the tissue regenerative capacity. Similar functional stem cell attrition has been observed in nearly all adult stem cell compartments, including the mouse forebrain, bones, and muscle fibers [60-62].

Stem cell attrition and the subsequent loss of functional stem cell reserves may arise from various mechanisms related to different hallmarks of aging, such as increased genomic instability and DNA damage burden, impaired DNA repair mechanisms, a disruption of proteostasis leading to the accumulation of misfolded proteins, epigenetic dysregulation, mitochondrial dysfunction, telomere attrition and telomerase inactivation, and the induction of cellular senescence programs. The convergence of these diverse insult compromises stem cell populations over time.

Recent studies have demonstrated that increasing the expression of cellular stress resistance factors such as the Hsp70 [63] or the forkhead transcription factor FOXO4, as well as pharmacological intervention with rapalogues such as rapamycin, can alleviate stem cell attrition and improve stem cell function during aging [64]. However, the precise mechanisms underlying such protective effects remain to be fully elucidated.

### 2.3.2 Altered intercellular communication

Another key integrative hallmark is the disruption of intercellular communication [65], which arises from age-related alterations in secreted proteins and signaling networks facilitating crosstalk between cells; in addition to cell-autonomous changes, senescence involves changes in the level of intercellular communication, whether endocrine, neuroendocrine, or neuronal [66-68].

Studies published in recent years have shown that glia direct axonal and synaptic pruning events. In the mammalian CNS, synapse removal and axon pruning are actively performed by at least two distinct glial cell types: astrocytes and microglia. Astrocyte-dependent synapse elimination may be a widespread event [69], and a disruption of microglial migration or their development leads to defective synaptic pruning in the hippocampus and cortex [70]. Processes such as chronic inflammation, deregulated nutrient sensing, and cellular senescence can perturb crucial signaling pathways governing tissue repair, immune function, and stem cell regulation within local microenvironments.

Thus, as we age, the ability of our immune system to assess pathogens and precancerous cells declines, our pericellular and extracellular environments change, and our inflammatory responses intensify. As a result, neurohormonal signaling (such as renin-angiotensin, adrenergic, and IIS signaling) tends to become dysregulated. An increase in the number of senescent cells that secrete proinflammatory cytokines (see the "Cellular senescence" section), the increased activation of NF-kB transcription factors, defects in autophagic responses, or the accumulation of tissue damage that causes inflammation are some of the factors that can cause inflammation [71]. The immune system's capacity to eliminate infections, diseased cells, and cells on the brink of malignant transformation may worsen immunological senescence by increasing the senescent phenotype throughout the body. Furthermore, the immune system recognizes and eliminates senescent cells (see the "Stem cell attrition" section) and pluripotent cells that accumulate in precancerous lesions and senescent tissues [72, 73].

Genetic, nutritional, or pharmaceutical therapies may enhance the aspects of intercellular communication that are lost with aging, providing several options for repairing damaged intercellular communication during senescence [74, 75]. Dietary restriction to extend healthy lifespan [76, 77] and rejuvenation strategies based on the use of blood-borne systemic factors identified in symbiosis experiments are of particular interest in this area [78-80]. Moreover, the regular use of anti-inflammatory medications such as aspirin may increase the lifespan of mice and promote good aging in humans [81, 82]. In addition, the human lifespan can be increased by adjusting the composition and function of the complex and dynamic gut microbiota, since the gut microbiome influences how the host immune system functions and affects systemic metabolism [83, 84].

### 2.4 Accumulation of substances

A certain accumulation of substances has been observed during aging, such as the localized accumulation of trace elements, iron and calcium, as well as proteins, free radicals and termination codon abnormalities.

Iron participates in mitochondrial oxidative phosphorylation, oxygen transport, and the formation of myelin, neurotransmitters, and DNA, among other critical functions in the nervous system [85]. Iron and ferritin levels were found to be associated with age-related increases in cerebellar, cortical and basal ganglia astrocytes; hippocampal and amygdala astrocytes; and microglia [85]. Additionally, in mice, iron levels were positively correlated with increased numbers of astrocytes in the mouse pallidum, striatum, and SN, whereas iron levels in the pallidum and striatum were also correlated with increased numbers of microglia [86].

Studies focusing on calcium levels have discovered distinct responses to increasing calcium levels with aging in the rat cortex, hippocampus, and cerebellar mitochondria (4-, 13-, and 25-month-old age groups) [87] and that sustained elevations in cytosolic calcium concentrations activate proteases, induce ROS production, and lead to neuronal apoptosis. Age-related alterations in calcium buffer regions may underlie the differential sensitivity to injury. Vulnerable neurons in individuals with AD, PD [35], and ALS [36] share the common feature of low levels of proteins that bind to calcium. Aging-related oxidative stress impairs ABCE1, leading to ribosome accumulation, and the ensuing cleavage of transcripts close to termination codons leads to the accumulation of 3'UTR [88] fragments during aging, which can lead to structural perturbations, the segregation of species within joint cells, and translational abnormalities followed by the aberrant expression of important proteins.

Additionally, the endoplasmic reticulum (ER) is significantly damaged with aging, leading to greater accumulation of protein abnormalities. ER stress progressively increases in the brain with aging, and delayed protein production and delivery greatly impair protein renewal. ER stress is also associated with protein degradation defects and toxic protein aggregation. These changes result in the accumulation of proteins such as hyperphosphorylated tau (p-tau), α-synuclein, human apolipoprotein E (ApoE), presenilin-1 (PS1) and presenilin-1 (PS2), amyloid proteins (APPs), β-amyloid (Aβ), and Huntington's protein (HTT) [89], and the accumulation of ROS because of the decrease in ROS scavenging capacity with aging, which makes aging neurons more vulnerable and creates a vicious cycle.

The majority of older adults exhibit significantly increased expression of age-related inflammatory markers, both in healthy individuals and in individuals with an illness or infection [90]. The accumulation of p-tau, α-synuclein, ApoE, APP, Aβ and other proteins is the main pathological manifestation of neurodegenerative diseases.

## 3. Aging and the neural microenvironment

Changes in the internal environment are particularly prominent in the aging process, with changes in the nervous and immune systems, in particular, serving as a focus of research. We will examine changes in the NVU, gray matter (GM) and white matter (WM) within the nervous system with aging. Regional heterogeneity in a number of age-related alterations has been observed in the nervous system, including alterations to the WM, GM, and vasculature of the brain and spinal cord [83].

### 3.1 Aging and the NVU

**Figure 2.**
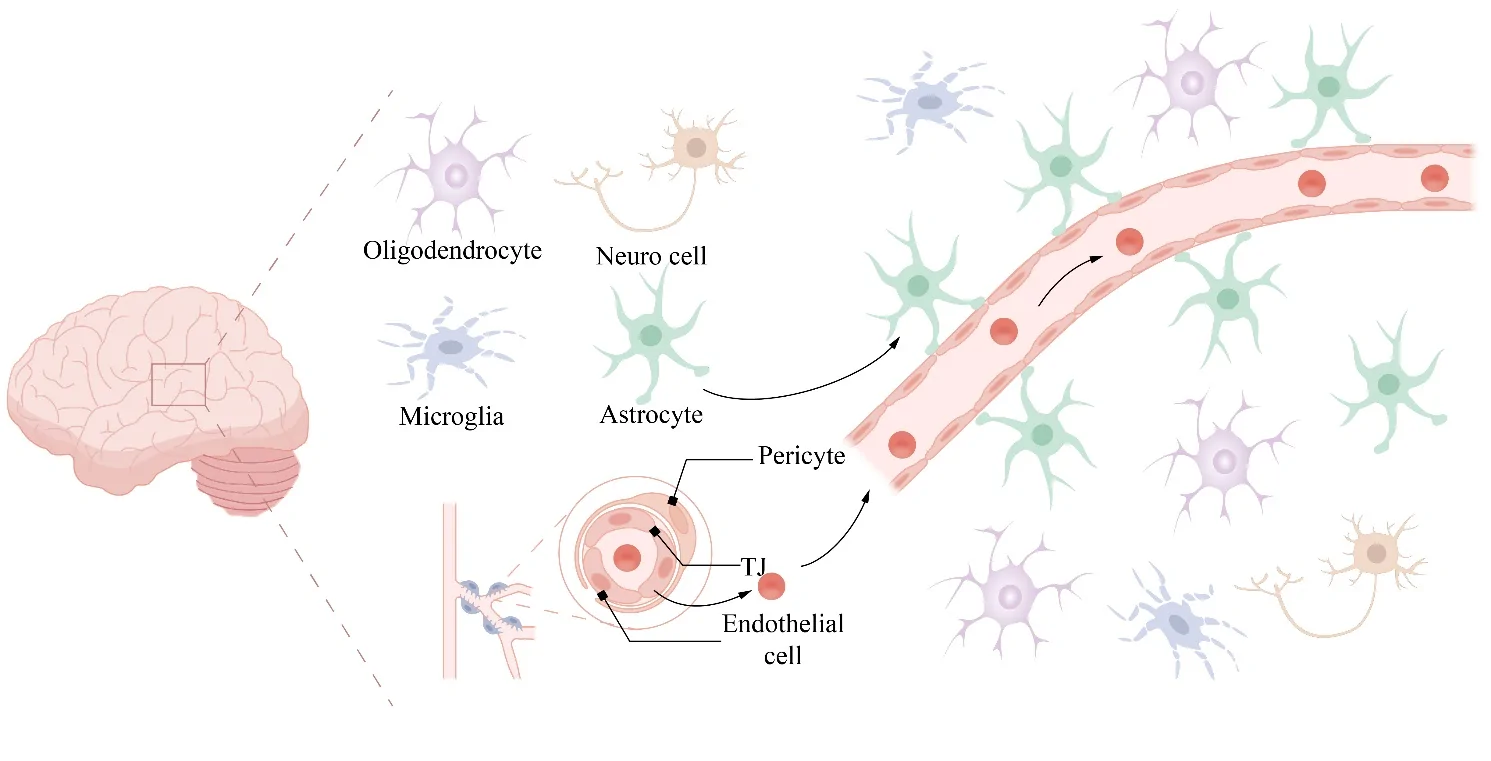
**The normal function of the brain is maintained by the NVU, which consists of a variety of neurons and glial cells, as well as vascular cells**. Among the glial cells involved are oligodendrocytes and microglia, and the vascular cells involved are endothelial cells, pericytes, smooth muscle cells, etc. The NVU is also the basis of the blood-brain barrier.

The NVU consists of neural progenitor cells and neurons, glial cells, vascular cells and the basal lamina matrix within the brain vasculature [91]. It also constitutes the blood-brain and blood-spinal cord barriers, which maintain homeostasis and prevents the entry of abnormal molecules (Fig. 2).

Most neurons throughout the CNS are nonrenewable. However, neurogenesis in the Dentate gyrus (DG) has been detected in specific regions, such as the subgranular zone (SGZ) and the subventricular zone (SVZ) [92, 93]. Nevertheless, the regenerative capacity of SGZ and SVZ neurons decreases in an age-dependent manner [94]. Axons extending from neurons in the CNS form a network of CNS neurons that are connected to the periphery for signaling and to receive excitatory substances, such as neurotransmitters, mainly glutamate.

The main types of glial cells are microglia, astrocytes, and oligodendrocytes. Astrocytes have the following functions: maintaining and regulating synaptic function (recycling neurotransmitters), releasing gliotransmitters, determining ionic homeostasis, and delivering nutrients to metabolically supportive neurons [95]. Astrocytes act as a link between the glial and vascular portions of the nerve in the NVU, supporting neuron-glia interactions. Additionally, during aging, abnormal protein accumulation has been observed in astrocytes in the laboratory as lipofuscin and intermediate filament bundle clustering [96].

Among the immune cells of the nervous system, microglia are primarily responsible for maintaining synaptic function, eliminating apoptotic neurons (their activation causes damage to mitochondria and death of neurons), and surveilling the surroundings to provide protection from pathogens. In response to invading pathogens or injury, normally quiescent microglia are activated, secrete various growth factors and cytokines and stimulate phagocytosis [97]. In senescent microglia, we detected a decrease in the expression of anti-inflammatory M2a markers, whereas the expression of proinflammatory M1 markers was relatively stable [98]. Both glial subtypes considerably contribute to the pathogenesis of ALS, PD, and AD [99-102]. On the other hand, oligodendrocytes produce lipid-rich myelin to coat axons and accelerate the conduction of impulses. Vascular cells are mainly endothelial cells, smooth muscle cells, and pericytes.

Tight junctions (TJs), which seal endothelial cells, pericytes, astrocyte endfeet, and the basal lamina, form the blood-brain barrier and maintain metabolic homeostasis in the nervous system [103, 104]. Among these factors, one of the primary contributors to impaired endothelial function is oxidative stress in smooth muscle cells and pericytes. In cells in the circulatory system, mitochondrial and ER dysfunction are key contributors to cellular impairment.

### 3.2 Aging and the GM/WM

The CNS consists of the GM and the WM, which exhibit different changes in volume with age in medical studies [105]. Researchers have divided the brain into 17 regions for study, broadly classifying the trends in the volumes of the GM and the WM as linear decreases; steep, nonlinear decreases; and stabilized postdecreases [106]. The GM volume increases with age in these regions, whereas the WM volume decreases overall, with a negative linear correlation with age in some regions and a nonlinear quadratic relationship with age in others [107]. The greatest rate of decrease in brain volume was observed in the 60-90-year-old age group, along with the greatest rate of reduction in the hippocampal volume [108].

The “last in, first out” hypothesis merits consideration, as it delineates a correlation between the temporal emergence of brain regions during individual development and phylogenetic progression and their propensity for early senescence. This finding suggests that WM fibers, which are the last to develop, are more vulnerable to age-related deterioration than those that develop earlier in the neurodevelopmental sequence [109].

Additionally, during aging, significant shrinkage of myelin sheets that isolate abyssal axons leads to slowing of nerve conduction [110]. Despite having a large energy requirement, the CNS has a very small energy storage capacity. In terms of cerebral blood flow (CBF), nearly all brain regions contain capillaries that provide sufficient energy and nutrition [111]. I In studies of neurovascular aging, areas of reduced CBF due to aging are associated with cognition and memory, whereas areas of lower arterial elasticity match areas with a smaller cortical volume, which is particularly likely to occur in areas of the WM (as opposed to the GM) and areas supplied by the anterior cerebral artery.

The hippocampus has also been shown to be a region susceptible to age-related decline in several studies [112, 113]. The different fiber types of the WM have been found to have different sensitivities to age-related changes, with a more pronounced decline in diffusion properties than in conjoined and projecting WM fibers [114]. Studies of fornix WM fiber tracts have revealed an anteriorposterior difference in sensitivity to aging, with the anterior fiber system, i.e., the geniculate, arachidonic, and fornix, being more susceptible to age-related changes than the posterior system (compression), and a greater tendency for the fornix to age. Since the hippocampus is where the fornix begins, the GM and WM in this area may be subject to aging [115]. Damage to the WM glial cells of the fornix during aging may, in turn, lead to hippocampal GM damage, which may predispose this region to increase the susceptibility of older adults to AD [116]. The fornix is more susceptible to aging than the posterior system (pressure), which is more susceptible to aging.

## 4. Aging and neurodegenerative diseases

According to World Population Prospects 2022, the population aged over 65 years is expanding more quickly than the population aged less than 65 years. By 2050, the proportion of the global population aged 65 years and over is projected to increase to 16 percent from 10 percent in 2022, while at the same time, neurodegenerative diseases, for which aging is a major cause, will have a major influence on aging societies. This demographic shift underscores the urgent need for effective interventions and therapies for age-related neurodegenerative disorders. Research into potential therapeutic targets for various neurodegenerative diseases has revealed a wide range of molecular mechanisms and pathways that may be amenable to intervention (Table 1). The subsequent sections elucidate the complex mechanisms through which aging influences the pathogenesis of various neurodegenerative disorders (Fig. 2). This analysis will draw upon insights gleaned from the studies summarized in Table 1, which highlight potential therapeutic targets across different neurodegenerative conditions.

### 4.1 Aging and AD

**Figure 3.**
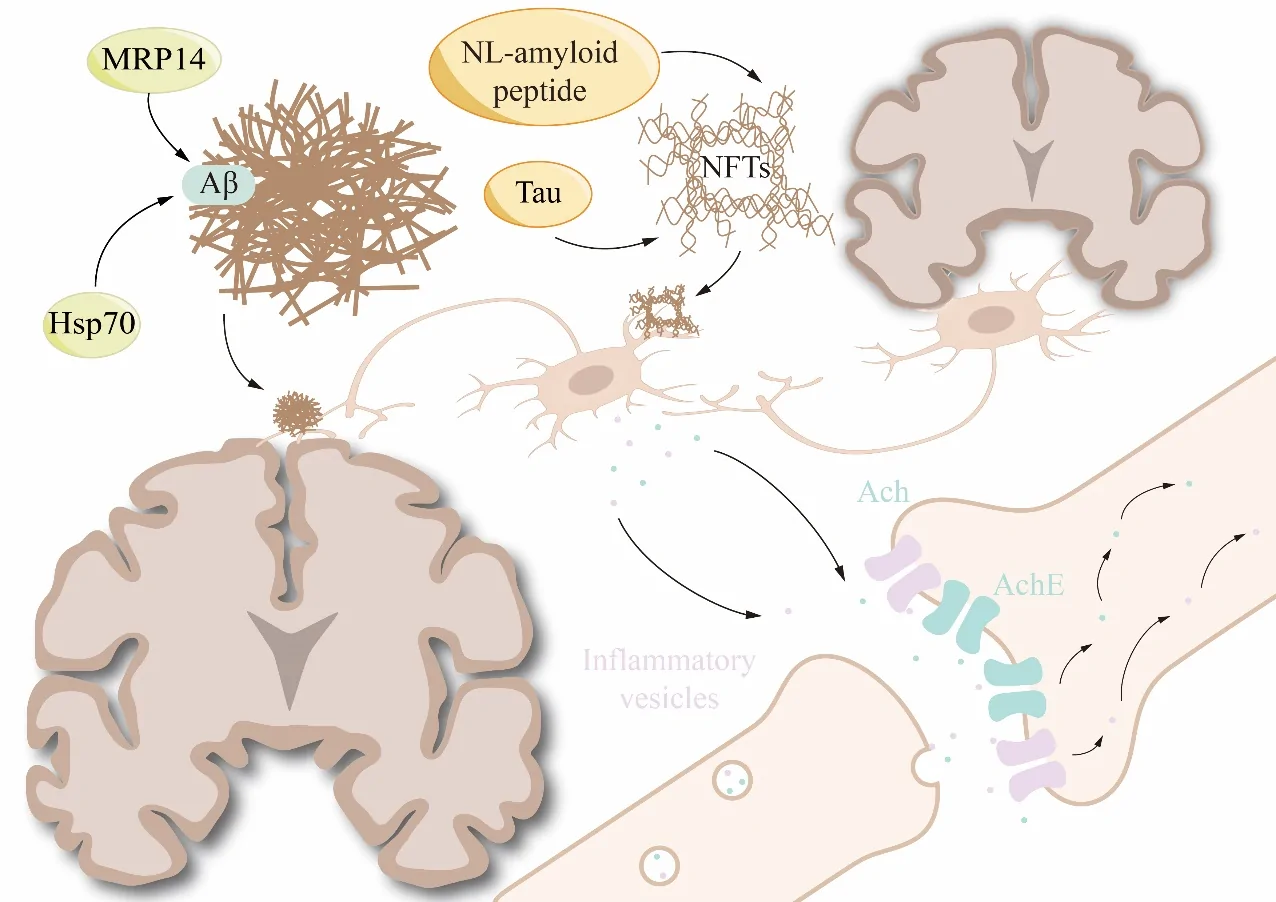
**Aging promotes Alzheimer's disease (AD) pathogenesis through multiple mechanisms: enhanced amyloid-beta (Aβ) aggregation, increased Tau hyperphosphorylation, chronic neuroinflammation, and cholinergic system dysfunction**. These age-related changes lead to extracellular plaque formation, neurofibrillary tangles (NFTs) accumulation, and elevated acetylcholinesterase (AChE) activity, ultimately resulting in neuronal damage and cognitive decline.

The primary symptoms of AD, the most prevalent neurological illness, include learning and memory impairments, mood swings, disorientation, and behavioral issues later in life. Its most prominent pathological manifestations are neuroinflammatory plaques, diffuse plaques, and neurofibrillary tangles (NFTs) [117]. Among the plaques, Aβ, which is associated with heat shock proteins (HSP) and the spinal cord-associated protein MRP14 (S100A9), activates innate immunity. NFTs are detected early in the internal olfactory cortex and hippocampus [118, 119]. The immune system is activated by tau/p-tau and NL-amyloid peptide, which are found in NFTs. The production of molecules that resist neurotoxicity and a highly active immune system can alter neurotransmitter levels and cause neuronal malfunction and death. Inflammatory vesicles, which are generated primarily by the elevated production of active caspase-1 in the brain, are a significant aspect of activation (Fig. 3).

**Table 1:** Potential therapeutic targets for age-related Neurological Diseases.

Diseases	Related targets	Study subject	Mechanism	Reference
**AD**	APP, PS1	Mouse	A slight decrease in DCX+ cells.	[247]
	Aβ	Mouse and NPCs	Aβ-treated NPCs decreased the expression of Ki67, GFAP, SOX2.	[248]
	Aβ	Rat	Aβ trigger spine loss by partially inhibiting NMDARs.	[249]
	PS2	Mouse	Deletion of PS2 does not affect hippocampal adult neurogenesis.	[250]
	ApoE	Human samples, mouse models	ApoE4 increases Aβ aggregation and impairs its clearance. ApoE-targeted therapies may reduce AD risk.	[251]
	Tau	Human samples	Tau accumulation occurs at different levels in the cortical areas of prodromal AD.	[252]
	PINK1(PARK6)	Mouse and NPCs	Increased apoptosis in PINK1-/- NSCs; Abnormal morphologic features of PINK1-/- DCX+ neurons.	[253]
	Parkin (PARK2)	Mouse	Arrested neuronal differentiation and abnormal morphology of NPCs; Decreased GFAP+ cells.	[254]
	Tau	Mice	Hyperphosphorylated tau forms neurofibrillary tangles.	[255]
	BDNF	Human samples	BDNF levels decrease with age and in AD. BDNF-mimetics may improve synaptic plasticity and cognitive function.	[256]
**ALS**	SOD1	SOD1-G93A mice	Reducing SOD1 levels or preventing its misfolding may slow disease progression.	[257]
	C9orf72	Patient-derived iPSCs	C9ORF72 repeat expansions lead to RNA toxicity and dipeptide repeat protein accumulation.	[258]
	TDP-43	TDP-43 transgenic mice	Decreasing TDP-43 homeostasis may be beneficial.	[259]
**PD**	α-synuclein	Human samples, animal models	Preventingα-synuclein aggregation or enhancing its clearance may be therapeutic.	[260]
	LRRK2	human embryonic kidney 293 cells overexpress-ing LRRK2	LRRK2 mutations increase kinase activity, leading to cellular dysfunction.	[261]
	Parkin/PINK1	Parkin/PINK1 knockout mice	Parkin/PINK1 pathway regulates mitophagy. Enhancing mitophagy may improve mitochondrial health in PD.	[254]
	GBA	Human clinical trials	GBA mutations impair lysosomal function and α-synuclein clearance.	[262]
**HD**	mHTT	Mouse	Reducing mHTT levels or preventing its aggregation may slow disease progression.	[263]
	BDNF	HD mouse models	BDNF-mimetics or increasing BDNF signaling may be neuroprotective.	[264]
**MS**	B-cell depletion	Human clinical trials	B-cell-depleting therapies reduce inflammation and disease activity.	[265]
	CD4+ T cell	Human clinical trial	Substantial CD4+ regulatory T cell responses were observed in all patients following immunization, accompanied by clinical improvement.	[266]
	NK cell	Human clinical trials	NK8+ cells can activate and proliferate CD4+ T cells in vitro.	[267]

Abbreviation: ApoE: Apolipoprotein E; BDNF: Brain-Derived Neurotrophic Factor; SOD1: Superoxide Dismutase 1; TDP-43: TAR DNA-binding Protein 43; LRRK2: Leucine-Rich Repeat Kinase 2; GBA: Glucocerebrosidase; mHTT: mutant Huntingtin.

The high heritability of AD, which is estimated to be in the range of 60-80% [120], is related to the ApoE gene [90], and the ApoE protein [121] is a major component of amyloid plaques and promotes Aβ aggregation and deposition. The lifetime risk of AD is more than 50% for ApoE4 homozygotes and 20-30% for heterozygotes for ApoE3 and ApoE4, whereas the overall risk is 11% for men and 14% for women, regardless of the ApoE genotype [122]. ApoE4 has multiple effects on AD. It interferes with the clearance of Aβ from the brain [123] and is processed into neurotoxic fragments [124].

The International AD Genomics Project expanded the list of susceptibility variations to more than 25 loci and conducted the largest cross-sectional case-control meta-analysis of AD diagnoses of persons using GWAS [120]. These risk factors that are also influential are age, nutritional and metabolic factors, and proteins. Age is the greatest risk factor [118] for AD, and studies have shown that the incidence of AD increases from 2.8 cases per 1,000 in the 65-69-year-old age group to 56.1 cases per 1,000 in the over 90-year-old age group.

Nutritional and metabolic factors mainly include insulin and IGF-1 resistance in the brain, which are common features of early AD. APP is the precursor of the Aβ peptides, and APP mutations impact Aβ cleavage and aggregation. The primary proteins include AE, APP, PS1, and PS2 [125]. Tau is also a diagnostic marker for AD, but mutations in the tau gene can cause dementia without amyloid plaques, i.e., tau proteins can act independently of Aβ [126]. However, in recent years, a concomitant decrease in the risk of dementia has been observed [127, 128], which may be an "off-target" effect of the ongoing improvements in prevention and management strategies for cardiovascular health, as well as improvements in the social determinants of health, including education.

Current treatments for AD include rapamycin, acetylcholinesterase inhibitors (donepezil, galantamine, and carboplatin), liraglutide, exercise, omega-3 fatty acids, curcumin, and flavanols. The FDA has approved acetylcholinesterase for use in patients with AD [118], and liraglutide [129] has been shown to prevent the decline of neurogenesis in the hippocampus in a mouse model.

### 4.2 Aging and ALS

ALS is characterized primarily by neurological and angiogenic deficits and widespread neuronal loss in the CNS, leading to progressive weakness, atrophy of random skeletal muscle, fibrillation and hypermobility of muscle bundles. Characteristic changes in upper and lower motor neurons involving the brainstem and multiple innervated parts of the spinal cord are the clinical hallmarks of ALS. Individuals may exhibit trunk or respiratory involvement (5%), limb involvement (approximately 70%), or medullary onset (approximately 25%) that eventually progresses to other areas [130]. Atypical presentations include emotional lability, rigidity and fasciculations (without muscle weakness), frontal lobe-type disorders of cognition, and weight loss (which indicates a poor prognosis) [131].

Because ALS is a progressive disease, 50% of patients die within 30 months after the onset of symptoms, and 20% live for five to ten years [132], which is worse than the prognosis of AD and PD [36]. Fatigue and a decreased exercise capacity are common symptoms of ALS [133]. Eventually, most patients require assistance with activities of daily living. Most patients with ALS develop dysphagia, and the ensuing weight loss and malnutrition are associated with a poor prognosis [134]. Most patients with ALS eventually develop respiratory compromise, resulting in exertional dyspnea, telangiectasia, hypoventilation with hypercapnia, and early-morning headache [135]. Once a patient develops dyspnea at rest, death is imminent.

In patients with ALS, the frontal cortex, lower brainstem, and ventral spinal cord all exhibit early increases in phosphorylated TDP-43 levels [136, 137]. As previously mentioned, the primary causes of ALS are alterations in the nucleocytoplasm, axonal structure and function, endosomal and vesicular transport, DNA repair, protein homeostasis, neuroinflammation, oligodendrocyte function and mitochondrial function.

Additionally, microglia and astrocytes as well as the activation of peripheral lymphocytes and phagocytes are the main pathological features of ALS, and NF-kB activation can activate astrocytes and induce neuronal death [137]. The use of the benzodiazepine receptor PET ligand [138] C-PK11195 revealed widespread microglial activation in individuals with ALS [139], which was supported by the discovery of inflammatory biomarkers in cerebrospinal fluid [140]. The incidence of ALS increases rapidly with age [141]. Most of the above hallmarks (including inflammation and autophagy) are associated with problems associated with aging, and the pathology of ALS increases with aging, leading to an exacerbation of its symptoms.

Several mechanisms of ALS have been well established through extensive research. The accumulation of protein aggregates, particularly TDP-43 [142], in motor neurons is a hallmark pathological feature observed in most ALS patients. Oxidative stress and mitochondrial dysfunction are also widely accepted as factors contributing to motor neuron degeneration. However, the exact triggers of these processes remain under investigation. The role of neuroinflammation [143], while consistently observed, is still debated as to whether it is a primary driver or a secondary response in ALS pathogenesis. Recent studies have suggested a potential gut-brain axis involvement in ALS, but this hypothesis requires further validation [144]. The contribution of environmental factors to ALS development is another area of active research, but definitive conclusions have not yet been reached.

Anderson's team [12] and Miller's team [145] have proven track records of successful clinical management of ALS patients. They have recommended therapeutic programs such as speech therapy, physical therapy, and certain medications for common ALS-related problems, such as weakness and disability, dysphagia, dyspnea and poor coughing, and pain (i.e., musculoskeletal pain and spasm, fascicular fibrillation and spasm, and painful skin pressure due to immobility).

### 4.3 Aging and MS

One of the most prevalent autoimmune illnesses in youth is MS, which is characterized by perivenous inflammatory lesions that eventually result in demyelinating plaques [146].

The inflammatory infiltrate contains T cells, predominantly MHC class I-restricted CD8^+^ T cells; B cells and plasma cells are also present [147]. The perivascular and parenchymal inflammatory infiltrate that enters the brain and spinal cord through the progressively permeable blood-brain and blood-spinal cord barriers is the initial stage of MS, which occurs when the adaptive immune system initiates an attack on the CNS. With the loss of the neural reserve, recovery from recurrence becomes incomplete, and neurological deficits progressively increase, leading to lasting disability [148]. Inflammation in MS is associated with the activation of immune cells, including T cells, B cells, astrocytes, microglia, and peripheral phagocytes, which release cytotoxic and proinflammatory cytokines and ROS. With aging, the body's ability to clear ROS decreases, and DNA damage increases, leading to the substantial accumulation of ROS. As mitochondrial autophagy decreases, the ability to control inflammation decreases, leading to the accelerated progression of MS due to inflammation. Patients with late-onset disease risk faster development of permanent disability than those with very young-onset disease, who only experience relapse and random events [149]. Patients with progressive MS are more likely to have slowly growing, chronic, active or smoldering (sometimes called mixed active and inactive) WM lesions, which peak at approximately age 50. Ongoing inflammation that is unfavorable for myelin repair develops in aged microglia [150].

The autoimmune nature of MS is well established, with the immune system attacking the myelin sheath of neurons. The role of T cells, particularly CD4+ [151] and CD8+ [152] T cells, in this process is firmly supported by evidence. The contribution of B cells [153] to MS pathology has garnered increasing recognition, leading to the development of B-cell-targeted therapies. However, the initial trigger for this autoimmune response remains elusive and is an area of ongoing research. The potential role of viral infections, particularly Epstein-Barr virus infection [153], in MS initiation has attracted significant attention but is still under investigation. While neurodegeneration is recognized as a key component of MS progression, especially in later stages, the mechanisms driving this process independent of inflammation are not fully understood. Recent hypotheses suggesting a primary neurodegenerative component in MS, alongside the inflammatory process, are gaining traction but require further validation.

### 4.4 Aging and HD

Medium-sized polyspinal neurons gradually disappear in patients with HD, an autosomal dominant neurodegenerative disease that begins in the striatum or extends into the cerebral cortex. HD can develop up to the age of 80 years, and before the onset of the disease, patients are healthy with no clinical abnormalities observed [154]. However, subtle changes in personality, cognition, and motor control may be detected [155].

During the progression of the disease, the individual may become irritable, uninhibited, and unreliable at work; multitasking becomes difficult, and forgetfulness and anxiety increase [156]. As the disease progresses, the affected individual demonstrates marked chorea, incoordination, motor nonpersistence, and slowed eye-sweeping movements. This disease is a major cause of the development of chorea [157]. Speech weakens more quickly than comprehension does. Cognitive dysfunction in patients with HD usually affects executive processes, such as organizing, checking, and planning. It also inhibits the development of new motor abilities.

Although inflammation is associated with microglia in the peripheral immune system [158], elevated plasma cytokine and chemokine concentrations and dysregulated monocyte and macrophage responses have been observed in the peripheral immune system [159]. In its cytotoxic form, HTT is linked to pathology, resulting in general cellular damage, including synaptic dysfunction and mitochondrial toxicity, which are linked to a decrease in the velocity of axonal transport.

Neurodegeneration in striatal medium spiny neurons may be attributed to increased glutamate release and a lack of synaptic glutamate clearance. In HD mouse models and human postmortem tissues, reduced expression of the astrocyte glutamate uptake receptors GLT1 and GLAST has been observed, which may explain the elevated glutamate levels [160, 161]. Excess synaptic glutamate levels are thought to lead to increased signaling by α-amino-3-hydroxy-5-methyl-4-isoxazole propionic acid (AMPA) receptors, postsynaptic N-methyl-D-aspartate (NMDA) receptors and kainate receptors. Consequently, this signaling may lead to calcium influx, chronic membrane depolarization, cell death pathway activation and oxidative stress [162-165].

The genetic basis of HD is firmly established: an expanded CAG repeat in the huntingtin gene is the definitive cause [166]. The toxicity of the resulting mutant huntingtin protein to neurons, particularly in the striatum and cortex, is well documented. However, the precise mechanisms by which mutant huntingtin leads to neuronal dysfunction and death are still unclear. While impaired protein degradation and transcriptional dysregulation are widely accepted as key pathogenic processes, the extent of their contributions to different aspects of HD pathology is still under investigation. The role of mitochondrial dysfunction in HD [167] has been consistently investigated, but whether it is a primary driver or a secondary effect remains a subject of debate. Recent hypotheses suggesting that HD might involve prion-like spreading of mutant huntingtin are intriguing but require further substantiation.

### 4.5 Aging and PD

PD is also a common neurodegenerative disease with symptoms such as neuromuscular dysfunction in the amplitude and velocity of movement, rigidity and/or resting tremor, and depression. The pathological manifestations include the progressive loss of dopaminergic neurons in the substantia nigra and the formation of neuronal cytoplasmic inclusions (Lewy bodies). Lewy pathology was first detected in the medulla oblongata and olfactory bulb [168, 169], followed by neuronal loss in the substantia nigra, leading to striatal dopamine deficiency and the formation of intracellular inclusions containing α-symnclein [170].

The greatest risk factor for PD is age, and the main risk genes are the location of the MAPT gene located to the tau gene, the p.R47H variant of the TREM2 gene, and pathogenic mutations in PSEN1 (p.A79V) and PSEN2 (p.V148l). Therefore, 5-10% of PD patients exhibit familial aggregation, with an estimated risk of genetically induced disease of up to 27%.

The main mechanisms of PD are altered cellular stress, organelle degradation, mitochondrial function, immune response, nutrition, and protein misfolding. Exosomes extracted from patients with PD contain increased levels of α-synuclein, and the inflammatory cascade associated with PD at the cellular level begins with the accumulation of misfolded α-synuclein, which plays a role in neuronal synapses. Microglia have been suggested to potentially play a crucial role in the delivery of α-synuclein via exosomes, while the proteostasis of α-synuclein has also been a focuse of research [171]. Mitochondrial autophagy, oxidative stress, calcium homeostasis, axonal transport, and neuroinflammation, which have been discussed in the context of the mechanisms underlying aging, also underlie the pathological development of PD.

Moreover, mTOR and AMPK targets [172] have previously been shown to play protective roles in PD. Two clinical approaches are used to treat PD: one involves inhibiting the SASP in senescent cells through the use of rapamycin^68^, melatonin [173], resveratrol [174], metformin, and estrogen [175]; the other is by inducing cell death.

## 5. Targeting aging to treat neurodegenerative diseases

Based on previous research on the mechanisms of aging and learning about neurodegenerative diseases, several interventions have been proposed [176], such as deep brain stimulation, antisense oligonucleotide supplementation, stem cell therapy, enhancement of the defense repair system, diet [177, 178], sleep [179], exercise [180, 181], rehabilitation, medications, and early education [176].

Commonly used medications include inhibitors of acetylcholine enzymes such as riluzole; inhibitors of mTOR [182] signaling in senescence (rapamycin and metformin), various antioxidants [183, 184] (e.g., resveratrol), anti-inflammatory medications [185] (nonsteroidal cumulative anti-inflammatory drugs and other anti-inflammatory medications, e.g., minocycline); antisense oligonucleotides [186]; elmination strategies involving levodopa, NAD^+^, and FOX04 peptides; BCL-W inhibitors; immunotherapy; and the induction of mitochondrial autophagy. Two main types of drugs induce mitochondrial autophagy. One type promotes mitochondrial autophagy by inducing mitochondrial damage, and due to their toxicity, these drugs are not suitable as human therapeutic agents. The second class promotes mitochondrial autophagy without disturbing mitochondria [187], such as urolithin A (Fig. 1).

Rehabilitation is one of the most important approaches for people with neurodegenerative diseases. Because natural aging results in diminished functionality for older people, a sedentary lifestyle, bed-riddenness or muscle wasting due to neurodegenerative diseases may lead to more physiological problems, such as muscular atrophy, a limited range of motion, decreased endurance and ultimately poor physical conditions. With natural aging, 20-40% of muscle strength is lost, which can lead to several disabilities [188], such as a decreased cardiorespiratory capacity. Proper exercise can play a crucial role in improving and preventing degenerative diseases [189], maintaining cognitive function [190], reducing depression, and improving quality of life. Some muscle strength (5-40%) can be restored through training [191]. The benefits of exercise also include neuroplasticity and the ability of the brain to repair itself [192].

Through cognitive training and motor training, the release of neurotrophic factors [193], protection of neurons, and remodeling of brain network connections are increased in patients, thus slowing the further deterioration of cognitive and motor functions and the progression of the disease. Moreover, targeted training can preserve and improve patients’ motor function (e.g., gait and balance), language and communication abilities, self-care ability and other daily living skills and maintain residual functions. Rehabilitation can also alleviate mental and behavioral disorders such as anxiety and depression, improve complications such as aphasia and dysphagia, and provide comprehensive symptom management such as respiratory training and nutritional support. Comprehensive intervention not only helps promote patients' integration into the community and self-efficacy but also improves their quality of life through optimal symptom management and psychological support.

Rehabilitation for neurodegenerative diseases encompasses a wide range of physical therapies beyond exercise. While exercise remains a cornerstone of treatment, other modalities play crucial roles in comprehensive care. Electrotherapy, including transcutaneous electrical nerve stimulation and functional electrical stimulation, has shown promise in managing pain and improving motor function in patients with conditions such as PD [194] and MS [195]. Thermotherapy, both in the form of heat and cold applications, can alleviate muscle stiffness and pain associated with various neurodegenerative conditions. Light therapy, particularly low-level laser therapy, has potential neuroprotective effects and may enhance mitochondrial function in AD and PD models [196]. Hydrotherapy leverages the properties of water to provide low-impact resistance training and improve balance, which is particularly beneficial for patients with mobility issues. Ultrasound therapy has been explored for its potential to enhance drug delivery across the blood-brain barrier and stimulate neuroplasticity. Magnetic stimulation, such as repetitive transcranial magnetic stimulation, shows promise in treating depression and potentially slowing cognitive decline in AD patients [197]. Additionally, virtual reality and augmented reality technologies are emerging as innovative tools for cognitive training and motor rehabilitation across patients with various neurodegenerative diseases. These diverse physical therapies, which are often used in combination and tailored to individual patient needs, form an integral part of the multidisciplinary approach to managing neurodegenerative diseases, complementing pharmacological treatments and lifestyle interventions.

Overall, rehabilitation, an important part of the comprehensive treatment of neurodegenerative diseases, plays a role in slowing the course of the disease, maintaining function, managing symptoms and improving quality of life. In conclusion, the focus of rehabilitation for different neurodegenerative diseases varies, but a multidisciplinary, holistic and integrated intervention model needs to be adopted to manage symptoms comprehensively, slow disease progression, maintain residual function and improve quality of life.

### 5.1 AD

AD patients exhibit cognitive impairment, behavioral abnormalities, emotional disorders, social function decline and other signs in the early stage of the disease. Comprehensive rehabilitation treatment, which can use physiotherapy to improve a patient's motor ability, maintain the patient's ability for daily life self-care through occupational therapy, and improve cognitive ability comprehensively through speech therapy, rehabilitation, recreational therapy and other treatments, should be administered in the early stage. For the treatment of psycho-behavioral symptoms, the main purpose is to influence the patient's psychology and behavior through speech, emotion, and behavior, through which the intervention can improve the patient's cognitive function and reduce psycho-behavioral symptoms. Psychomotor therapists in France work with patients to accomplish this intervention with the psycho-behavioral support of health insurance [198]. Many studies have also been conducted on new treatments for Alzheimer's disease, but the results thus far have been disappointing [199-207].

Rehabilitation for AD focuses on cognitive and functional training, aimed at slowing down cognitive decline and delaying the loss of daily living skills. Behavioral interventions are also needed to manage behavioral symptoms, and exercise training is needed to prevent physical decline and osteoporosis.

AD drug development has emerged as one of the most dynamic and promising areas in neuropharmacology. While traditional treatments such as acetylcholinesterase inhibitors (e.g., donepezil, rivastigmine, and galantamine) and the NMDA receptor antagonist memantine [208] continue to play crucial roles in symptom management, the field is rapidly evolving with several novel approaches. These approaches include amyloid-targeting therapies such as monoclonal antibodies (e.g., aducanumab, lecanemab, and donanemab) [209], tau-targeted treatments [210], anti-inflammatory approaches [211], neuroprotective strategies [212], and combination therapies [213]. The FDA's approval of aducanumab [214] in 2021 marked a significant milestone as the first disease-modifying therapy for AD. Additionally, precision medicine initiatives are exploring treatments tailored to patients with specific genetic or biomarker profiles. Despite challenges in translating preclinical success to clinical efficacy, the diverse AD drug development pipeline reflects the complexity of AD pathology and the need for multifaceted treatment strategies. This burgeoning field provides hope for more effective treatments and potentially disease-modifying therapies in the near future, underscoring the importance of continued research and investment in AD drug development.

### 5.2 ALS

The clinical drug generally used to treat ALS is riluzole [215], but edaravone is an antioxidant agent ; thus, oxidative stress is likely the primary mechanism of action and requires free radical scavenging [216], which was approved by Canada, the FDA, Japan and other Asian countries. Antioxidant agents are well known to prevent aging. Additionally, the FDA recently approved AMX0035 as a medicine [217], which has been used to reduce mitochondrial dysfunction and ER stress. In contrast, the effect of masertinib [218] on ALS is experimental.

Despite the lack of a cure for ALS [219], treatments focus on supportive therapies, such as respiratory training to delay respiratory muscle weakness, nutritional support to ensure nutrient intake, functional training to maintain self-care and communication with assistive devices, and psychosocial support [220].

### 5.3 MS

Currently several pharmacological treatments are available for this condition, including immunosuppressants (fingolimod, natalizumab, and orolizumab) [221] and immunomodulators (interferon beta, teriflunomide, glatiramer acetate, and teriflunomide), which require ongoing treatment to suppress inflammation and disease activity [148]. Symptomatic treatment of the disease, such as medications for symptoms caused by neurological damage or physiotherapy, such as anticholinergic medications for bladder dysfunction (which are medications for neuropathic pain (usually tricyclic antidepressants or gabapentin and its derivatives)), may also be taken. If patients have comorbidities, their original survival rate and prognosis are reduced [24].

The main goal of rehabilitation for MS patients is to maintain and improve various functions, reduce primary symptoms, and reduce or delay the onset of functional disability. For example, passive joint mobility training and plyometrics are used to improve joint function and maintain muscle strength. A patient's level of fatigue should be considered, which may be alleviated by cold therapy. MS onset reduces the brain's cognitive reserve, and exercise and a healthy diet are recommended. In studies of leukocyte telomeres, we also reported that telomeres in patients with progressive MS are shorter than those in their healthy counterparts [222].

### 5.4 HD

Currently, chorea, mental, and cognitive symptoms are primarily managed as part of HD treatment. The FDA has approved tetrabenazine and deutetrabenazine for the treatment of HD, since they have been shown to successfully diminish chorea in HD patients [223, 224]. Neuroprotective effects of inhibiting the mTOR pathway were observed in HD cells and animal models in response to mTOR inhibition. As a result of rapamycin administration, mutant huntington aggregates and neuronal atrophy are reduced in Drosophila and mouse models of HD [225]. Additionally, rapamycin can be used in combination with lithium to treat certain conditions. Lithium activates a mechanism independent of mTOR to induce autophagy by inhibiting inositol monophosphatase. This dual approach appears to have a cumulative effect on the HD fly model [226]. Metformin, another antagonist of mTOR, reduces the aggregation of HTT mutants and decreases early behavioral deficits in a mouse model of HD [227]. Metformin was linked to improvements in cognitive performance in HD patients, without discernible changes in motor function [228].

The primary focus of the current treatment for HD is on managing symptoms. Rehabilitation of patients with HD requires a holistic approach that addresses the full range of motor, cognitive, psychiatric, and communicative symptoms. The focus includes motor training to improve coordination and balance, cognitive training to slow cognitive impairment, behavioral interventions to address emotional and behavioral problems, and speech training to alleviate communication disorders.

### 5.5 PD

The main treatment for PD is anti-parkinsonian drugs, including levodopa, madopa and cirelin. The maintenance of brain plasticity by dopamine analogs maintains existing neural pathways.

Rehabilitation for PD patients [229] currently focuses on training for tremors, myotonia, bradykinesia, and postural balance disorders, as well as the prevention of secondary dysfunctions that arise as a result. Rehabilitation for PD patients is based on exercise training to improve motor deficits such as gait, balance and mobility. As an adjunct and complementary therapy, it improves cortical striatal plasticity and increases dopamine release. Exercise training is effective at in improving motor deficits (including balance, gait, fall risk, and physical functioning) and nonmotor deficits (such as cognitive function, sleep disturbances, and quality of life) [230] in PD patients.

Functional training preserves self-care, and cognitive behavioural therapy relieves nonmotor symptoms such as anxiety and depression. Sensory (auditory, visual, and proprioceptive) feedback can be used to help PD patients relearn normal postures because of the abnormal postures they often have. PD patients are prone to fatigue, which slowly disappears after it occurs. Therefore, first, the patient should avoid resistance activities. Second, the patient with PD should be instructed to learn strength-saving techniques and let the patient use these strength-saving techniques in daily life to minimize the impact of the disease. Last, the patient should also be taught relaxation training to let him reduce muscle fatigue due to the muscle ankylosis caused by the PD. Moreover, psychological problems caused by the disease also require psychotherapeutic interventions to help patients reduce the vulnerability caused by the disease.

## 6. Brain reserve

Early education, such as education, leads to structural changes in the brain, such as a large brain volume, surface area and curvature [231-234]. Recall that the "brain reserve" hypothesis, in which the effects of education on structural cognitive functioning of the brain are primarily established during childhood/adolescence neuro-development and are largely retained into old age, is not based on neuroprotective effects on cognition/brain aging. Individuals with higher levels of education may have an initial advantage in brain reserve, and this advantage persists into adulthood/senescence [235]. Rather than diminished total wanting changes in brain structure and cognition, this process reduces the risk of individuals with higher levels of education being diagnosed with AD (i.e., the interception effect of education across the lifespan) [236].

Studies have shown that for every 4.2 years of education, the odds of developing AD are reduced by approximately 30% [231]. Sleep, physical activity, smoking, alcohol consumption, cognitive leisure activities, diet and meditation, which have been studied as effective interventions for neurodegenerative diseases, are equally effective interventions for cognition.

## 7. Conclusions

A critical examination of neurodegenerative disease research, particularly AD, research reveals significant controversies and conflicting findings. The ongoing debate surrounding the amyloid hypothesis in AD, conflicting views on disease progression mechanisms in Parkinson's disease, and discrepancies in understanding the role of neuroinflammation in ALS highlight the complex landscape of this field. These conflicts may arise from methodological differences, disease heterogeneity, publication bias, replication challenges, translational gaps, and technological limitations. Recent instances of research misconduct, such as potential image manipulation in influential AD papers, have further complicated the field, potentially misdirecting resources and eroding public trust. These issues underscore the critical need for more rigorous peer review processes, independent replication of key findings, and greater data transparency. While deeply concerning, these challenges present an opportunity for researchers in the field to strengthen their practices, ultimately improving the quality and reliability of neurodegenerative disease research. Moving forward, the research community must remain vigilant and self-critical, fostering an environment that prioritizes scientific integrity and robust, reproducible findings. This approach will be essential for advancing our understanding of these complex disorders and developing effective therapies.

These incidents underscore the critical importance of scientific integrity and the need for the research community to remain vigilant and self-critical. Moving forward, more rigorous peer review processes are clearly needed, particularly for high-impact studies that may influence the direction of the field. Greater emphasis should be placed on independent replication of key findings before they are widely accepted or used as the basis for clinical trials. Encouraging data transparency and diversifying research approaches can help prevent overreliance on single hypotheses or research groups. While deeply concerning, these incidents present an opportunity for researchers in the field to strengthen their practices, ultimately improving the quality and reliability of AD research and restoring public confidence in the scientific process.

This critical examination extends beyond AD to other neurodegenerative diseases as well. Across the field, we must address limitations in current research methodologies, such as the challenges in translating findings from animal models to human patients, the difficulties in conducting long-term studies for slowly progressing diseases, and the complexities of capturing the heterogeneous nature of these disorders. Furthermore, we must critically evaluate the sensitivity and specificity of current biomarkers and diagnostic tools, especially for early disease stages. By acknowledging these limitations and actively working to overcome them, we can increase the robustness and reliability of neurodegenerative disease research, paving the way for more effective treatments and interventions.

Through this comprehensive review, we systematically characterized the multiple mechanisms underlying aging and their intricate connections to neurological impairment. Primary aging hallmarks, such as genomic instability, telomere attrition, epigenetic alterations, and proteostatic disruptions, initiate a cascading decline in cellular and molecular homeostasis. The progressive accumulation of damage across multiple physiological domains, including stem cell exhaustion, cellular senescence, deregulated nutrient sensing, and loss of proteostasis, is a fundamental driver of the gradual functional deterioration observed during healthy aging.

Strikingly, many of the same biological processes that are disrupted in normal aging also constitute central pathological nodes in various neurodegenerative disorders. Pathological manifestations such as oxidative stress, mitochondrial dysfunction, neuroinflammation, and the accumulation of misfolded proteins represent downstream effects that are exacerbated in disease states such as PD, AD, and ALS. Rather than being distinct entities, aging and neurodegeneration can be conceptualized as a continuum, where pathological conditions accelerate and amplify the deleterious effects stemming from the aging process itself.

This revised mechanistic framework, which identifies aging as a prime risk factor and driver of neurological diseases, provides a unifying perspective to guide therapeutic development. Interventions that beneficially modulate core aging pathways at various levels, including pharmacological approaches targeting specific molecular mechanisms synergized with multidomain lifestyle modifications such as exercise, CR, and cognitive engagement, may achieve enhanced preventative and restorative effects. Combination strategies harnessing the crosstalk between different hallmarks could yield synergistic benefits. Recent studies on TLR9 inflammatory signaling have also demonstrated that maintaining the integrity of TLR9 inflammatory signaling is a promising preventive strategy for neurocognitive deficits.

The biological underpinnings of aging and neurodegenerative pathologies are closely intertwined, with the same core processes being perturbed to different extents. This holistic reconceptualization paves an exciting path forward. By elucidating and therapeutically targeting the common hallmarks shared between aging biology and neurodegenerative pathologies, we can extend not only lifespan but also health span for the increasing elderly populations worldwide more comprehensively.

Several critical research avenues emerge for future scientific inquiry given the multifaceted and intertwined nature of the aging process: elucidating core regulatory nodes, delineating context-specific aging triggers, developing synergistic multitarget therapies, and harnessing novel therapeutic modalities. While major aging hallmarks such as decreased autophagy and mitochondrial dysfunction have been identified, additional mechanisms should be delineated to identify key regulatory nodes governing these processes. The application of systems biology approaches and multiomics profiling could reveal crucial upstream drivers and controller hubs within the aging network. Precise molecular mapping is vital for rationally designing interventions.

Different cell types, tissues, and environmental contexts may activate distinct initiating factors that propagate aging cascades. Systematically investigating these triggers and their complex interplay will optimize the timing of therapeutic windows. Longitudinal studies tracking individuals from preclinical stages are warranted. Identifying early aging insults enables preemptive countermeasures before irreversible damage can accumulate.

Given the intertwined nature of aging mechanisms, multipronged interventions that simultaneously modulate several pathways may achieve enhanced preventative and restorative effects compared with single-target approaches. Combination regimens combining senolytics [237], autophagy inducers [238], anti-inflammatories [239] and mitochondrial nutrients represent promising options. Leveraging AI for multicomponent optimization could accelerate drug development.

In addition to small molecules [240], emerging modalities such as gene therapies [241], mRNA technologies [242], and novel biomaterials [243] hold the potential for more precise aging interventions. Targeted gene editing of longevity pathways, in vivo reprogramming toward youthful epigenetic states, and biomaterial-mediated delivery of rejuvenating signals are innovative strategies that should be explored.

Emerging technologies are poised to revolutionize the treatment of age-related neurodegenerative diseases. Gene therapy and editing techniques, such as CRISPR-Cas9 [244], offer unprecedented potential for correcting genetic risk factors or introducing neuroprotective genes. Stem cell therapies [245], present opportunities for cell replacement and neural tissue regeneration. These cells can be differentiated into specific neural cell types, such as dopaminergic neurons for PD treatment or oligodendrocytes for promoting remyelination in multiple sclerosis.

Advanced neuroimaging techniques, including high-resolution PET scans with novel tracers, are enhancing early diagnosis and treatment monitoring. These imaging modalities can detect pathological changes in the brain long before clinical symptoms appear, potentially allowing for earlier intervention. For instance, amyloid and tau PET imaging have revolutionized the diagnosis and monitoring of AD progression. Additionally, nanotechnology [246] is enabling targeted drug delivery to the brain, potentially overcoming the blood-brain barrier challenge. Nanoparticles can be engineered to carry therapeutic agents across this barrier, increasing drug concentrations in the brain while minimizing systemic side effects. This approach could dramatically improve the efficacy of existing drugs and enable the use of promising compounds that were previously limited by poor brain penetration. Moreover, nanotechnology offers the potential for creating multifunctional platforms that combine drug delivery with diagnostic capabilities, paving the way for more personalized and effective treatments for neurodegenerative diseases.

In parallel, computational and modeling advancements are accelerating research and drug discovery. Artificial intelligence and machine learning algorithms are accelerating the identification and efficacy predictions of the efficacy of potential therapeutic compounds. The development of "brain-on-a-chip" and 3D organoid has provided models offering more accurate representations of human brain physiology for drug testing and disease modeling. These cutting-edge approaches hold the promise of more personalized, effective treatments, although they face challenges in safety, ethics, and clinical validation. As these technologies mature, they may fundamentally transform our ability to prevent, treat, and potentially reverse neurodegenerative processes, providing new hope in the field.

This review has analyzed the impact of aging on neurological diseases in the elderly, focusing on molecular mechanisms and neural microenvironment. We examined aging's role in neurodegenerative diseases such as Alzheimer's, Parkinson's, Huntington's, ALS, and MS. However, limitations exist in our analysis. The complexity and heterogeneity of these diseases, coupled with genetic and environmental variability, make definitive conclusions challenging. Moreover, most studies reviewed rely on animal models and in vitro experiments, which may not fully represent human aging processes.

Future research should address these limitations through more extensive human studies, particularly longitudinal investigations. Developing sophisticated biomarkers and personalized medicine approaches could offer promising avenues for treatment. Additionally, exploring the role of lifestyle factors in mitigating aging's effects on the brain is crucial. In conclusion, while our understanding has advanced, much remains to be learned. Continued research in human clinical trials, biomarker development, personalized medicine, and lifestyle interventions will be vital in improving therapeutic strategies for neurodegenerative diseases in the elderly. By addressing these challenges, we can hope to make substantial progress in combating age-related neurological disorders and enhancing the quality of life for our aging population.

In summary, deciphering the intricacies of aging biology through multidisciplinary and systems-level approaches will catalyze the development of novel preventative paradigms for age-related disorders. Coupled with cutting-edge therapeutic platforms, these insights pave the way toward extending the healthspan of increasing elderly populations worldwide.

## Data Availability

All data in this review are publicly available.
